# Clinical Evaluation of Antiphospholipase A2 Receptor IgG4 level and Its IgG4-to-IgG Ratio Based on Quantitative Immunoassays in Idiopathic Membranous Nephropathy

**DOI:** 10.1155/2022/9127520

**Published:** 2022-05-13

**Authors:** Yi Zhang, Yiqing Huang, Biao Huang, Xiaolei Chen, Bin Zhou, Pei Zou, Liang Wang, Xiaobin Liu, Huiming Sheng, Minhao Xie

**Affiliations:** ^1^NHC Key Laboratory of Nuclear Medicine, Jiangsu Key Laboratory of Molecular Nuclear Medicine, Jiangsu Institute of Nuclear Medicine, Wuxi 214063, China; ^2^Department of Nephrology, The Affiliated Wuxi People's Hospital of Nanjing Medical University, Wuxi 214023, China; ^3^College of Life Sciences and Medicine, Zhejiang Sci-Tech University, Hangzhou 310018, China; ^4^Tongren Hospital, Shanghai Jiao Tong University School of Medicine, Shanghai 200336, China; ^5^Department of Radiopharmaceuticals, School of Pharmacy, Nanjing Medical University, Nanjing 211166, China

## Abstract

**Background:**

Phospholipase A2 receptor (PLA2R), located at the membrane of glomerular podocyte, is the major autoantigen of idiopathic membranous nephropathy (IMN), and its antibodies with a predominant IgG4 subclass lead to pathological lesions. Further studies could be performed to validate the clinical values of PLA2R-IgG, PLA2R-IgG4, and PLA2R-IgG4-to-IgG ratios, as ultrasensitive and quantitative immunoassays for PLA2R antibodies have been well established in our previous work.

**Methods:**

A cohort of 58 IMN patients, 30 of whom were followed from 3 to 42 months, was assessed for serum PLA2R-IgG and -IgG4 levels, and the ratio of PLA2R-IgG4/-IgG combined with relative clinicopathological indicators.

**Results:**

Serum PLA2R-IgG4 level was significantly correlated with glomerular PLA2R staining. In addition, it was strongly correlated with PLA2R-IgG and its ratio. PLA2R-IgG and -IgG4 levels were both correlated with high-density lipoprotein and erythrocyte sedimentation rates. The ratio at the first diagnosis can predict the remission, and its efficacy overmatched PLA2R-IgG4. In the survival curves, negative results for the ratio or PLA2R-IgG4 at the first diagnosis demonstrated higher remission rates.

**Conclusion:**

Serum PLA2R-IgG4 concentration may replace renal PLA2R immunohistochemistry in IMN diagnosis. We propose that the PLA2R-IgG4-to-IgG ratio and PLA2R-IgG4 could be novel indicators for remission prediction in clinical practice.

## 1. Introduction

Membranous nephropathy (MN) is an autoimmune kidney disease that is a common cause of nephrotic syndrome in adults, and its incidence has increased gradually in children over the past decades [1–4]. It is characterized by massive proteinuria (>3.5 g/day) and diffuse thickening of the glomerular basement membrane, due to the presence of immune deposits [1, 5]. Approximately 80% of MN cases are idiopathic MN (IMN) with target antigens and circulating antibodies for the immune complex, and the rest are secondary MN associated with other systemic diseases or drug exposures [6–8].

The major antigen in IMN is the M-type phospholipase A2 receptor (PLA2R), a transmembrane protein present in human podocytes [9]. PLA2R is identified as a 185 kD protein that belongs to the mannose receptor family [10]. Antibodies against PLA2R are found in most patients, making a breakthrough in diagnosing and monitoring the disease without renal biopsy in certain situations. Their detection is now being used as a promising tool for predicting the tissue diagnosis of PLA2R-associated MN among patients with preserved kidney function without evidence of secondary causes [5]. For anti-PLA2R antibody analysis, serum anti-PLA2R IgG is generally detected by immunoassays [11–13]. Human IgG has four subclasses, of which PLA2R-IgG4 is predominant in patients with IMN. However, serum PLA2R-IgG4 levels could not be accurately determined until 2019 [11, 14]. Therefore, through the absolute quantification of serum PLA2R-IgG and PLA2R-IgG4 levels, a thorough understanding of the PLA2R-IgG4 level and PLA2R-IgG4/-IgG ratio is necessary for the diagnosis and treatment of IMN.

This study assessed serum PLA2R-IgG, PLA2R-IgG4, and their ratio using quantitative immunoassays with other pathological and clinical indicators for IMN before and after treatment. We evaluated their clinical usefulness and prognostic value in patients with IMN at a local center.

## 2. Materials and Methods

### 2.1. Patients

A total of 58 IMN patients proved by renal biopsy were enrolled in this study in the Affiliated Wuxi People's Hospital of Nanjing Medical University. Among all, 30 patients were followed up for at least 3 months. This study adhered to the Declaration of Helsinki and was approved by the Ethics Committee of the Affiliated Wuxi People's Hospital of Nanjing Medical University (KYL2016001). The patients had signed informed consent.

### 2.2. Data Collection

Specimens and basic data from 58 patients were collected prior to treatment, of which 30 patients received immunosuppressive therapy and continued sampling during follow-up. The basic features presented including gender, age, and with or without a history of hypertension. The pathological indicators included the total score of renal tubular and interstitium examined by light microscopy, immunofluorescence (IF), and electron microscopy, MN stage classified according to Ehrenreich and Churg standards, and IF PLA2R considered positive or negative according to glomerular staining of PLA2R protein [15]. Laboratory characteristics included serum C3, IgG, albumin (ALB), creatinine (Scr), estimated glomerular filtration rate (eGFR, calculated using the Chronic Kidney Disease Epidemiology Collaboration equation), blood urea nitrogen (BUN), glucose (Glu), uric acid (UA), total cholesterol (TC), triglycerides (TG), low-density lipoprotein (LDL), high-density lipoprotein (HDL), white blood cell (WBC), hemoglobin (HB), platelet (PLT), erythrocyte sedimentation rate (ESR), hematuria, and 24 h proteinuria. All laboratory tests were conducted in accordance with standard operating procedures. Serum anti-PLA2R IgG and its IgG4 subclass were quantitatively detected by time-resolved fluoroimmunoassay developed by Jiangsu Institute of Nuclear Medicine. The PLA2R-IgG4/-IgG ratio was calculated by dividing PLA2R-IgG4 by PLA2R-IgG.

### 2.3. Definitions

Hypertension was defined as systolic blood pressure ≥ 140 mmHg, diastolic blood pressure ≥ 90 mmHg, or with antihypertensive drugs. Treatment efficacy was classified as complete remission (CR), partial remission (PR), and no remission (NR). CR was determined when urinary protein was <0.3 g/d with stable eGFR. PR was achieved when proteinuria was 50% reduced to a level < 3.5 g/d. While NR was considered when proteinuria did not arrive at the guideline for PR. The reference ranges in the manufacturer's manuals were 0-25/*μ*L for hematuria, 0-0.3 g/24 h for proteinuria, 8.6-17.4 g/L for serum IgG, 40-55 g/L for ALB, 0-1.7 mmol/L for TG, 0-5.18 mmol/L for TC, 0-3.37 mmol/L for LDL, and 0-15 mm/H for ESR. The cut-off ranges for PLA2R-IgG, PLA2R-IgG4, and PLA2R-IgG4/-IgG ratio were 0-1990 ng/mL, 0-161.2 ng/mL, and 0-8%, respectively.

### 2.4. PLA2R-IgG and PLA2R-IgG4 Determinations

The PLA2R-IgG and PLA2R-IgG4 assays were described as follows [12, 14]. A 100 *μ*L of PLA2R-IgG standard or serum sample (diluted 1 : 200 in assay buffer) was pipetted into each well of a microtiter plate coated with the recombinant PLA2R antigen. After 1 h of incubation at 25° C, the plate was washed three times and then added with 100 *μ*L of diluted Europium-labeled goat anti-human IgG antibody in the assay buffer. After another 1 h of incubation at 25° C, the plate was rinsed six times and pipetted with 100 *μ*L of enhancement solution. The plate was agitated for five minutes and read using an autoDELFIA_1235_, a time-resolved fluoroimmunoassay instrument. The fluorescence intensity was recorded and the PLA2R-IgG concentration of each sample was calculated by the instrument system. The PLA2R-IgG4 determination was the same as the above protocol but pipetted with the sample diluted 1 : 20 and Europium-labeled mouse anti-human IgG antibody in the first and second incubation, respectively.

### 2.5. Statistical Analysis

Statistical analyses were performing with SPSS 22.0 (SPSS Inc., Chicago, IL, USA) and Prism 8.0 (GraphPad, San Diego, CA, USA). The measurement data were judged on the basis of whether they obeyed a normal distribution using the Shapiro-Wilk test. If they were, they were expressed as mean ± standard deviation, and differences between the two groups were compared using the independent samples *T* tests. If not, they were presented as the median, 25th and 75th percentile, and two-group differences using the Mann–Whitney *U* test. Enumeration data were analyzed by the Chi-square test. The Spearman correlation coefficient was used to calculate correlations between the variables. The linear correlation was analyzed by the Pearson's test. The 1-survival curves were plotted for the remission and analyzed by the Kaplan-Meier method and the Breslow-Wilcoxon test. A two-tailed *P* value < 0.05 was considered significant statistically.

## 3. Results

### 3.1. Basic Characteristics of IMN Patients and Comparisons with and without PLA2R Antigen

**Figure 1:**
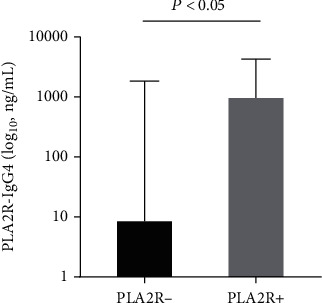
Serum PLA2R-IgG4 levels in the PLA2R-negative and PLA2R-positive groups.

A total of 58 IMN patients with a median age of 55 years were from MN stage I-II and classified into IF PLA2R negative and positive groups. The general characteristics of the patients were listed in Table 1. Fifty-one subjects (87.9%) were positive for the PLA2R antigen, and seven (12.1%) were negative. Thirty-seven of the 58 subjects (63.8%) were also considered to have hypertension. The median or mean serum IgG, ALB, TG, TC, LDL, ESR, PLA2R-IgG, PLA2R-IgG4, and PLA2R-IgG4/-IgG ratios were abnormal according to the reference ranges. Only serum PLA2R-IgG4 levels were significantly increased in the IF PLA2R-positive group, whereas the other clinical and pathological characteristics were not significantly different. The PLA2R-IgG level and PLA2R-IgG4/-IgG ratio, with hematuria and Scr, tended to increase in IF PLA2R-positive patients but was not statistically significant (*P* > 0.05). The serum C3 and IgG levels tended to decrease in the PLA2R-positive group. The median of serum PLA2R-IgG4 was 9.0 and 1016.2 ng/mL in the PLA2R-negative and PLA2R-positive groups, respectively (Figure 1).

### 3.2. Correlations between PLA2R Antigen or PLA2R Antibodies and Clinical Indicators

Clinicopathological parameters were compared by the Spearman correlation test with IF PLA2R antigen, the PLA2R-IgG and PLA2R-IgG4 levels, and the PLA2R-IgG4/-IgG ratio. As listed in Table 2, PLA2R antigen on the glomerular basement membrane was significantly correlated with the level of serum PLA2R-IgG4 (*P* < 0.05) and the PLA2R-IgG4/-IgG ratio (*P* < 0.05) compared to serum PLA2R-IgG (*P* = 0.11) in IMN patients. Serum levels of PLA2R-IgG and PLA2R-IgG4 tended to increase with ESR and decrease with HDL levels. Moreover, PLA2R-IgG4 was strongly correlated with PLA2R-IgG and PLA2R-IgG4/IgG ratio. A linear correlation was significant between PLA2R-IgG4 and PLA2R-IgG with *R* = 0.70 and *P* < 0.01. The linear regression equation was *y* = 1312.3 + 1.9*x*.

### 3.3. Clinicopathological Characteristics of IMN Patients with and without Remission

**Figure 2:**
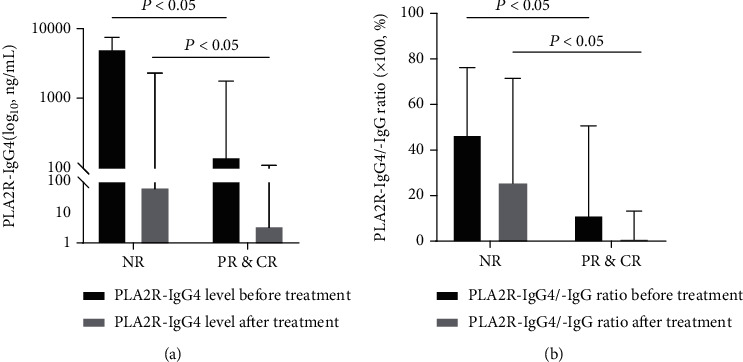
Comparison of PLA2R-IgG4 levels (a) and PLA2R-IgG4/-IgG ratios (b) in IMN individuals with or without remission. The data from before and after treatment were presented as a box chart.

Among the 58 patients enrolled in the study, 30 received treatment and were followed up. After a follow-up period of 3 to 42 months, 9 subjects had NR and 21 subjects achieved PR or CR according to the Kidney Disease Improving Global Outcomes guideline [5]. Clinical indicators before and after therapy were listed and compared in Table 3. The median follow-up periods were 3 and 6 months for the nonremission and remission groups, respectively. Compared with NR patients, PR or CR patients exhibited much lower levels of proteinuria, serum PLA2R-IgG4, and PLA2R-IgG4/-IgG ratio with higher ALB after the therapy. The PLA2R-IgG tended to be lower and eGFR was higher at the baseline in the remission group. The level of serum PLA2R-IgG4 and the ratio of PLA2R-IgG4/-IgG detected before therapy were significantly lower than those in the NR group. In summary, high PLA2R-IgG4 levels and PLA2R-IgG4/-IgG ratios were high-risk factors for IMN remission (Figure 2). However, the descender of all characteristics except proteinuria, represented as Δ in Table 3, showed no significant difference from the baseline.

### 3.4. Correlation of Clinical Factors and Remission

Twenty patients in remission, including PR and CR, were diagnosed according to proteinuria levels.  Table 4 summarized the clinical parameters according to remission status. Proteinuria and ALB after treatment were strongly correlated with remission (*r* = −0.91, *P* < 0.01; *r* = 0.77, *P* < 0.01). Compared with the PLA2R-IgG, the PLA2R-IgG4/-IgG ratios at baseline and endpoint were negatively related to remission (*P* < 0.05). PLA2R-IgG4 levels after treatment were also negatively related to outcome (*P* < 0.05). However, there was no correlation or significant difference between the nonremission and remission groups with other common clinical indicators.

### 3.5. Associations of PLA2R-IgG4 and PLA2R-IgG4/-IgG ratio with Remission Outcomes

**Figure 3:**
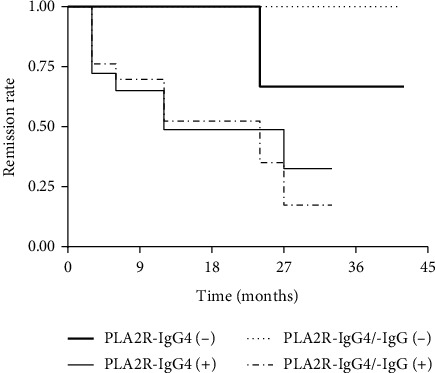
Kaplan-Meier curves of overall survival for partial and complete remission based on the detection of serum PLA2R-IgG4 and PLA2R-IgG4/-IgG before treatment in 30 IMN cases.

Figure 3 plotted the overall remission according to the administration of PLA2R-IgG4 level and PLA2R-IgG4/-IgG ratio with 42 months of maximum follow-up. Patients with negative PLA2R-IgG4 level or PLA2R-IgG4/-IgG ratio had a higher remission rate than the positive ones, while PLA2R-IgG4/-IgG-negative individuals had a relatively higher remission rate than PLA2R-IgG4-negative ones. The Breslow-Wilcoxon test showed that after a median follow-up of 12 months, there was a significant difference in PLA2R-IgG4 levels between the negative and positive groups (*P* < 0.05), indicating that a low serum PLA2R-IgG4 would have a large effect on remission in early treatment.

## 4. Discussion

In this study, we described some of our main findings. Compared with anti-PLA2R IgG, the serum level of IgG4 subclass was significantly increased and correlated with renal PLA2R antigen-positive IMN patients. This relationship was also evident between PLA2R-IgG4/-IgG ratio and PLA2R antigen. In addition, PLA2R-IgG4 was strongly associated with PLA2R-IgG and PLA2R-IgG4/-IgG. Anti-PLA2R IgG and IgG4 subclass were both positively related to ESR and negatively relevant to HDL. In follow-up studies, remission patients exhibited lower PLA2R-IgG4 and PLA2R-IgG4/-IgG levels at the baseline, and significantly higher ALB and lower proteinuria, PLA2R-IgG4 and PLA2R-IgG4/-IgG after treatment. The PLA2R-IgG4-to-IgG ratio was negatively related to PR or CR and more sensitive than PLA2R-IgG4. Furthermore, consistently PLA2R-IgG4-negative patients achieved remission relatively quickly at the initial follow-up.

Since Dahnrich et al. first developed enzyme-linked immunoassays for the determination of PLA2R-IgG and IgG4 subclasses [11], several studies have promoted the understanding of antibodies against PLA2R [16–20]. Here, we identified the importance of IgG4 and IgG4/IgG of anti-PLA2R in IMN. Compared with PLA2R-IgG, PLA2R-IgG4 was found to be a more efficient biomarker for predicting the risk of progression in IMN. With a high sensitivity of >98% [14], PLA2R-IgG4 significantly decreased with remission of proteinuria after immunosuppressive therapy. In this Chinese cohort, continuous PLA2R-IgG4 negativity would predict PR or CR achievement. However, the levels for PLA2R-IgG4 and PLA2R-IgG detection utilized relative units, which resulted in the limitation of assay accuracy, being unable to obtain the ratio of the subtype 4, and even could not determine the relationship between PLA2R-IgG4 and other subclasses. Here, based on our previous work [12, 14], we presented the levels of PLA2R antibodies in ng/mL as the measurement unit and simply obtained the ratio of PLA2R-IgG4/-IgG by division. The PLA2R-IgG4/-IgG ratio, first assessed for its clinical value and therapeutic effectiveness, was found to be associated with the prediction of IMN treatment outcomes. The consistently lower level of PLA2R-IgG4/-IgG with PLA2R-IgG4, would promise a better recovery.

Human IgG can be divided into IgG1, IgG2, IgG3, and IgG4 subclasses, wherein IgG4 corresponds to anaphylactogen as the least abundant in plasma. However, anti-PLA2R IgG4 is predominant, followed by IgG1, IgG3, and IgG2, in IMN [9, 19]. With accurate quantification of PLA2R antibodies, the ratio was significantly higher in the PLA2R-positive group, which indicated that IgG4 was the main subclass of PLA2R antibodies and could injure the glomeruli by recognizing the PLA2R antigen. Patients with a low PLA2R-IgG4/-IgG ratio at first diagnosis easily achieved remission, which indicated that the other subclasses except IgG4 played an import role in IMN pathogenesis. It proves that IgG subclasses switch during disease progression [21]. The decreasing ratio in remission patients proved that IgG4 was not consistently dominant in all pathological stages, suggesting that the IgG1-3 subclasses participated in the remission process. This finding may explain the different proportions of serum IgG1-3 subtypes in IMN patients from another side [19]. More studies are needed on IgG1-3 anti-PLA2R to elucidate the physiopathologic mechanism of IMN.

In our study, the PLA2R-IgG4 and PLA2R-IgG4/-IgG levels, but not PLA2R-IgG levels, were positively associated with glomeruli PLA2R-positive patients, implying that the IgG4 subclass mainly participated in the complex formation of the glomerular basement membrane in IMN, as IgG4 can be found in immune deposits with PLA2R antigen on renal tissue [9, 22]. This study also verified that PLA2R-IgG4 is associated with proteinuria in the pathological mechanism of IMN, which further indicated that PLA2R-IgG4 impaired renal function. Moreover, PLA2R-IgG4 significantly correlated with PLA2R-IgG (*r* = 0.7, *P* < 0.05), which was similar to the results of a previous study. These findings indicate that the detection of serum PLA2R-IgG4, but not PLA2R-IgG, could reduce the rate of renal biopsies.

From this cohort, we found that HDL was negatively associated with PLA2R-IgG4 and PLA2R-IgG. This might explain why lipid and lipid-related proteins play a major role in modulating podocyte function. Glomerular disease is associated with an APOL1 sequence variant, which encodes apolipoprotein L1, expressed in podocytes, and an important component of HDL. Fetal HDL does not have antioxidative functions without apolipoprotein L1 [23]. Moreover, Larsen et al. reported that the presence of APOL1 risk alleles serves as an accelerating factor in MN [24]. As a type of nephrotic syndrome, IMN patients suffer from metabolic syndromes, such as hyperlipemia even with hypertension. It clarified that in these patients, TG and TC were higher than the reference ranges of clinical and laboratory parameters, LDL was slightly higher, and most of whom had hypertension.

ESR provides adjective information for the clinical observation of a patient's changes in clinical condition, disease progression, and treatment effect. In renal diseases, it was observed that a high ESR was correlated significantly with persistent proteinuria, tubulointerstitial injury scores, poor long-term outcomes, and urine IgG levels [25–29]. We confirmed that a high ESR was commonly found in IMN and was relative to high PLA2R-IgG and PLA2R-IgG4 levels in serum plasma.

The present study has some limitations. We conducted a single-center study with an insufficient sample size, which may have affected the significance of the tests. Furthermore, each patient would be followed up every 3 months and well documented in future studies. A prospective study with a larger sample size would be required to promote the better understanding of the PLA2R-IgG4/-IgG ratio in the diagnosis and surveillance in the treatment of MN.

## 5. Conclusion

In conclusion, serum PLA2R-IgG4 and PLA2R-IgG4/-IgG ratio were more strongly correlated with glomerular PLA2R antigen than serum PLA2R-IgG in IMN patients, and the PLA2R-IgG4-to-IgG ratio was significantly and negatively associated with partial or complete remission. Patients with negative PLA2R-IgG4 levels may have higher remission rates during the first 12-month treatment. Therefore, PLA2R-IgG4 could be a noninvasive marker for confirmation and therapy of IMN, and the importance of the PLA2R-IgG4/-IgG ratio and PLA2R-IgG4 level should be considered in predicting the clinical outcome of IMN treatment.

## Figures and Tables

**Table 1:** Baseline clinical and pathological characteristics in IMN patients with and without PLA2R antigen.

Characteristics	Total	PLA2R -	PLA2R +	*P* value
IMN patients (*n*, %)	58	7 (12.1)	51 (87.9)	—
Age (years)	55 (42-64)	66 (39-76)	55 (42-64)	0.30
Male (*n*, %)	35	5 (71.4)	30 (58.8)	0.69
Hypertension (*n*, %)	58	4 (57.1)	33 (64.7)	0.69
Total score of renal tubular and interstitium	3 (2-5)	3 (2-5)	3 (2-5)	0.40
MN stage (*n*, %)	1 (1-2)	1 (1-1.5)	1 (1-2)	0.77
Hematuria (/*μ*L)	57.1 (22.7-104.9)	18.4 (16.2-400.7)	57.7 (26.0-104.9)	0.16
Proteinuria (g/24 h)	5.5 ± 2.4	5.8 ± 1.8	5.4 ± 2.5	0.69
C3 (mg/L)	895.2 ± 271.5	1037.7 ± 276.1	879.2 ± 263.3	0.13
IgG (g/L)	6.1 (4.1-9.1)	7.2 (4.3-12.8)	5.9 (4.5-8.5)	0.23
ALB (g/L)	20.6 ± 6.2	21.2 ± 4.5	20.5 ± 6.3	0.92
Scr (*μ*mol/L)	79.8 ± 24.3	68.6 ± 18.0	80.1 ± 24.8	0.25
eGFR (mL/min/1.73m^2^)	89.4 ± 24.6	97.1 ± 19.7	89.8 ± 25.2	0.38
BUN (mmol/L)	4.7 (3.7-6.1)	5.2 (4.6-8.7)	4.7 (3.5-6.1)	0.28
Glu (mmol/L)	5.0 (4.7-5.3)	5.1 (4.6-5.7)	5.0 (4.7-5.3)	0.35
UA (*μ*mol/L)	347.1 ± 87.2	353.4 ± 94.5	344.4 ± 86.5	0.82
TG (mmol/L)	2.3 (1.5-3.0)	2.4 (1.9-2.5)	2.3 (1.4-3.3)	0.84
TC (mmol/L)	7.0 (5.7-8.3)	7.0 (5.0-8.3)	6.7 (5.6-8.3)	0.64
LDL (mmol/L)	3.5 (2.8-4.7)	3.9 (2.7-5.0)	3.5 (2.8-4.7)	0.77
HDL (mmol/L)	1.3 ± 0.4	1.4 ± 0.4	1.3 ± 0.4	0.54
WBC (×10^9^/L)	6.3 (5.5-7.7)	6.8 (5.4-9.4)	6.3 (5.5-7.7)	0.97
HB (g/L)	123 (116-137)	137 (109-149)	122 (114-135)	0.71
PLT (×10^9^/L)	221.2 ± 67.8	200.9 ± 34.2	223.6 ± 69.5	0.45
ESR (mm/H)	45 (28-82)	50 (25-71)	45 (28-90)	0.97
PLA2R-IgG (ng/mL)	2326.3 (550.9-6055.8)	659.9 (266.2-2757.7)	2399.8 (604.3-7453.6)	0.11
PLA2R-IgG4 (ng/mL)	859.6 (30.3-3868.2)	9.0 (6.1-1840.4)	1016.2 (112.1-4250.0)	0.04
PLA2R-IgG4/-IgG (%)	41.4 (2.5-71.5)	2.6 (0.9-63.7)	43.5 (5.0-72.6)	0.18

**Table 2:** Spearman correlation analyses between PLA2R antigen/anti-PLA2R antibodies level and clinicopathological variables.

Variables	IF PLA2R		PLA2R-IgG level		PLA2R-IgG4 level		PLA2R-IgG4/-IgG level	
	*r*_*s*_	*P* value	*r*_*s*_	*P* value	*r*_*s*_	*P* value	*r*_*s*_	*P* value
Age	-0.14	0.30	0.19	0.15	0.13	0.33	-0.05	0.72
Gender	0.08	0.53	-0.15	0.27	-0.18	0.18	-0.13	0.32
Hypertension	0.05	0.70	0.11	0.40	0.20	0.14	0.15	0.25
Total score	-0.12	0.37	-0.04	0.74	0.04	0.77	-0.05	0.72
MN stage	0.11	0.41	-0.10	0.47	0.02	0.86	0.17	0.19
IF PLA2R	/		0.21	0.11	0.27	0.04	0.18	0.17
Hematuria	0.19	0.15	0.12	0.38	0.09	0.50	-0.07	0.59
Proteinuria	-0.03	0.81	0.12	0.39	0.05	0.70	-0.07	0.59
C3	-0.17	0.21	-0.05	0.71	-0.10	0.46	-0.09	0.52
IgG	-0.16	0.23	0.09	0.50	0.03	0.81	0.02	0.91
ALB	-0.03	0.81	-0.22	0.10	-0.16	0.22	0.04	0.76
Scr	0.15	0.26	0.08	0.53	0.15	0.28	-0.03	0.81
eGFR	-0.13	0.33	-0.14	0.30	-0.18	0.17	-0.01	0.94
BUN	-0.15	0.28	-0.01	0.94	-0.02	0.87	-0.09	0.52
Glu	-0.13	0.34	0.15	0.27	0.20	0.13	0.21	0.11
UA	0.04	0.77	-0.11	0.40	-0.06	0.67	0.03	0.84
TG	0.03	0.84	0.17	0.21	0.24	0.08	0.10	0.47
TC	0.07	0.63	-0.02	0.90	-0.04	0.75	-0.12	0.37
LDL	0.04	0.76	-0.20	0.14	-0.21	0.12	-0.13	0.34
HDL	-0.09	0.53	-0.37	0.01	-0.42	<0.01	-0.26	0.06
WBC	-0.01	0.97	-0.22	0.10	-0.25	0.06	-0.22	0.10
HB	-0.05	0.71	-0.13	0.34	-0.10	0.47	0.02	0.91
PLT	0.12	0.37	-0.08	0.56	-0.09	0.51	-0.07	0.63
ESR	-0.01	0.96	0.28	0.04	0.38	<0.01	0.23	0.10
PLA2R-IgG	0.21	0.11	/		/		/	
PLA2R-IgG4	0.23	0.04	0.81	<0.01	/		/	
PLA2R-IgG4/-IgG	0.31	0.02	0.13	0.33	0.71	<0.01	/	

*r*_*s*_ is the Spearman correlation coefficient.

**Table 3:** Clinical characteristics in IMN patients with and without remission.

Clinical characteristics	NR	PR or CR	*P* value
Age	59.2 ± 16.4	59.7 ± 13.8	0.94
Male (*n*, %)	5 (55.6)	13 (61.9)	0.53
MN stage	1.5 (1.0-2.0)	1.5 (1.0-2.0)	0.86
ALB (g/L)			
*b*	17.3 ± 6.7	21.4 ± 6.5	0.14
*a*	20.1 ± 4.4	33.2 ± 6.5	<0.01
Δ (%)	15.6 (-5.4 ~ 60.6)	54.9 (29.8-75.9)	0.07
Proteinuria (g/24 h)			
*b*	6.8 ± 1.3	5.1 ± 2.1	0.04
*a*	4.8 (3.8-6.1)	0.7 (0.2-1.7)	<0.01
Δ (%)	-17.1 (-39.5 ~ -11.4)	-84.2 (-95.9 ~ -57.6)	<0.01
Scr (*μ*mol/L)			
*b*	86.6 ± 25.3	80.3 ± 22.2	0.50
*a*	77.8 ± 16.7	76.4 ± 20.2	0.86
Δ (%)	-14.5 (-33.5 ~ 19.4)	-7.8 (-17.3 ~ 11.5)	0.50
eGFR (mL/min/1.73 m^2^)			
*b*	72.4 ± 23.2	86.6 ± 19.7	0.10
*a*	85.7 ± 25.2	88.4 ± 19.6	0.75
Δ (%)	20.9 (-4.0 ~ 49.0)	4.5 (-9.2 ~ 17.9)	0.15
PLA2R-IgG (ng/mL)			
*b*	8005.4 (1614.5-16797.5)	2741.4 (375.6-10440.0)	0.33
*a*	303.1 (166.4-5263.4)	410.7 (340.8-665.1)	0.82
Δ (%)	-89.3 (-97.7 ~ 18.7)	-77.9 (-97.0 ~ -12.3)	0.69
PLA2R-IgG4 (ng/mL)			
*b*	4906.0 (727.0-7517.4)	134.8 (30.7-1752.9)	0.01
*a*	67.6 (9.1-2293.5)	3.4 (1.0-107.7)	0.02
Δ (%)	-83.5 (-99.7 ~ -11.1)	-94.8 (-99.4 ~ -81.6)	0.66
PLA2R-IgG4/-IgG (%)			
*b*	46.2 (29.3-76.2)	10.8 (5.2-50.6)	0.01
*a*	25.3 (3.2-71.5)	0.7 (0.3-13.2)	0.01
Δ (%)	-10.2 (-90.8 ~ 40.1)	-73.0 (-97.6 ~ -9.2)	0.26
Time (mo)	3 (3-18)	6 (3-9)	0.59

NR: no remission; *b*: before treatment; *a*: after treatment, Δ: *a*/*b* − 1.

**Table 4:** Spearman's correlation between remission and clinical indicators.

Indicators	Remission	
	*r*_*s*_	*P* value
Age	0.07	0.73
Gender	-0.10	0.59
Total score	0.03	0.89
MN stage	0.16	0.41
ALB (g/L)		
*b*	0.28	0.14
*a*	0.77	<0.01
Δ (%)	0.38	0.04
Proteinuria (g/24 h)		
*b*	-0.36	0.05
*a*	-0.91	<0.01
Δ (%)	-0.88	<0.01
Scr (*μ*mol/L)		
*b*	-0.21	0.28
*a*	-0.08	0.70
Δ (%)	0.20	0.28
eGFR(mL/min/1.73 m^2^)		
*b*	0.25	0.18
*a*	-0.02	0.93
Δ (%)	-0.37	0.05
PLA2R-IgG (ng/mL)		
*b*	-0.07	0.73
*a*	0.07	0.70
*Δ* (%)	0.03	0.90
PLA2R-IgG4 (ng/mL)		
*b*	-0.28	0.13
*a*	-0.45	0.01
Δ (%)	-0.23	0.22
PLA2R-IgG4/-IgG (%)		
*b*	-0.38	0.04
*a*	-0.53	<0.01
Δ (%)	-0.34	0.06
Time (mo)	0.24	0.21

*r*_*s*_: Spearman correlation coefficient; *b*: before treatment; *a*: after treatment; Δ: *a*/*b* − 1.

## Data Availability

The data generated and analyzed during the present study are available from the corresponding authors on reasonable request.
